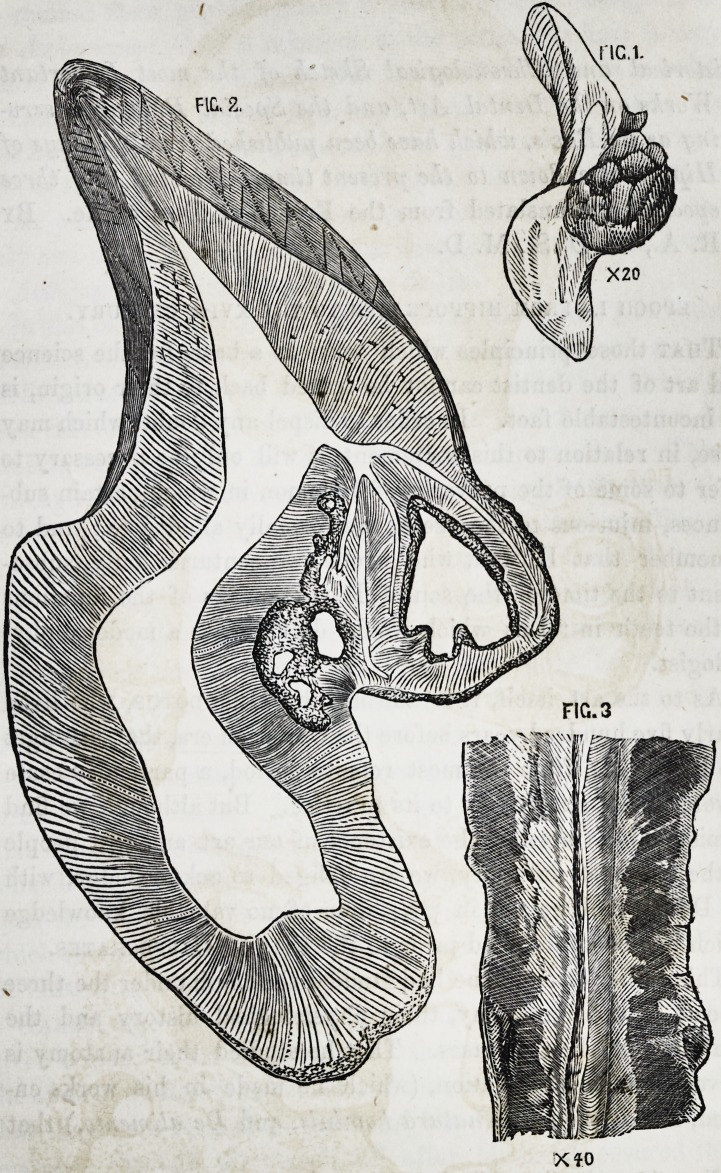# Papers on Dental Pathology

**Published:** 1857-01

**Authors:** S. James A. Salter


### ARTICLE II.

*Papers on Dental Pathology.* By S. James A. Salter, M. B., F. L. S.*

### 1. *Dentine-excrescence within the Pulp-cavity of an Incisor Tooth, which had been apparently associated with Neuralgia.*

The association of neuralgic pains of the jaws and face with exostosis on the surface of the fangs of teeth, pressing on the nerves of the alveolar periosteum, is a frequent occurrence, and is very generally known; but neuralgia, in conjunction with that form of secondary dentine, called *dentine-excrescence*,†

* Mr. Salter having kindly furnished the senior editor with the following highly interesting papers on dental pathology, taken from the transactions of the Pathological Society for 1855, we take pleasure in giving them to the readers of the Journal.—Eds.

† I have elsewhere enumerated and divided the several forms of *Secondary Dentine* into "Osteo-dentine," "Dentine of Repair," and "Dentine Excrescence." The first (*Osteo-dentine*) is produced by intrinsic calcification of the within the pulp-cavity, pressing on the nerves of the tooth-pulp, has, I believe, never been described—a circumstance which is possibly due rather to the imperfect investigation which the diseases of the teeth have received at the hands of pathologists, than to its infrequency.

The following history and the accompanying figures are illustrative of an example of this condition.

During the past year a woman applied to me at Guy's Hospital, on account of severe neuralgic pains obviously connected with one of the central incisors of the upper jaw. The pain was described as of a gnawing character, abiding, but not constantly severe; frequently amounting to a mere consciousness of the presence of the tooth, and at other times, sharp and darting. In the former condition it was confined to the region of the tooth; in the latter it flashed up the side of the face, and through all the branches of the superior maxillary division of the fifth nerve of that side. Sudden pressure, or a tap upon the tooth, or a marked change of temperature, produced a considerable augmentation of pain. The tooth itself was sound, to all external appearance; it was somewhat elongated beyond its fellow, and was very slightly loose. The gum surrounding it was red at the edge, and a little swollen.

This tooth had never received a blow, nor was there any known circumstance to account for the symptoms which it appeared to originate. When the tooth was removed no exostosis was discovered on it, and, with the exception of small patches of half-organized lymph, it appeared quite healthy.

Upon making a section of the tooth (vertical, from side to side,) I found an oval pear-like excrescense of dentine growing from the side of the pulp-cavity, so as to encroach much upon tooth-pulp, resulting in a conversion of the pulp into little systems of dentine, each surrounding a separate blood-vessel. *Dentine of Repair* consists of circumscribed patches of dentine laid on in the pulp-cavity, bulging into it, and in direct structural continuity with dentinal tubes which have been injured at their external abutment, either by fracture, wear or decay. *Dentine Excrescence* consists of nodules of secondary dentine, more or less adherent to the primary tissue, not permeated by blood-vessels, nor materially adherent to the pulp, nor obviously associated with lesion of the tooth.—"Guy's Hospital Reports," vol. viii, part 2.

it, and occupying, for a short space, more than half its diameter. It was of an oval form, its long axis corresponding to that of the tooth; in color less opaque, and yellower than the neighboring tissue, (Plate I, Fig. 1.)

In general appearance this nodule of secondary dentine resembled, not a little, a mass of dentine of repair; there was the same general convex surface in the pulp-cavity; but it differed in this, that its basal attachment was constricted, so as to give it, when seen in section, a somewhat mushroom-shaped form. Its difference from dentine of repair is more clearly shown by microscopical scrutiny.

A thin, accurately made section of this mass displays its singular structural composition; and viewed with a low power of the microscope (40 diameters, as in Plate I, Fig. 2,) its general arrangement can be comprehended at once. The section of the excrescence resembles in outline the head of a mushroom without its stalk, the central third of its basal surface being attached to the primary dentine. The dentinal tubes radiate from the centre of the attachment, but with a somewhat irregular and tortuous course. They are crossed at right angles by a series of laminæ.

The dentinal tubes of the excrescence appear to take their origin from the primary dentine at a small distance in its substance—the abnormal and irregular tubes radiating from that point, and the collateral tubes of the primary dentine being divergent, so as to leave a triangular interval within which the projecting base of the secondary tissue protrudes. From this it would seem that the excrescence commenced its formation before the tooth had arrived at mature adult growth. The tubular communication between the primary and secondary tissue is very circumscribed, for though the attachment of the excrescence occupies one-third of its basal surface, it has a far less extent of tubular continuity, for, at the line of attachment, the canals of the latter so converge, and those of the former so diverge, that they do not anastomose, excepting deeply, at the centre. The other two-thirds of the basal surface are not attached, but are separated by an obvious line of demarcation. All this will be better understood by reference to the figure.

In all these respects this structure differs from dentine of repair; for the latter tissue always (at least in every specimen I have seen) adheres over its entire base—which, too, is its broadest part—to the primary tissue, and the structural continuity throughout is most obvious; in fact the dental tubes can readily be traced at every point from one to the other. Dentine of repair more resembles, in the uniformity and evenness of its structure, normal primary dentine, than any other variety of secondary dentine.

The specimen now under discussion exhibits, to a marked degree, many of those irregularities in the structure, course, and form of the dental tubes, which are of frequent occurrence in the different varieties of secondary dentine. And though these are common to all three varieties of the secondary tissue, and indeed, to a certain extent, occasionally met with in primary dentine, they are here, nevertheless, so marked that they constitute a striking feature of the specimen.

The tubes of this excrescence are to a great extent filled up with secondary deposit within them—a condition common to all forms of the secondary tissue, and likewise found in primary dentine that has been subject to inflammation—in all cases associated with hypernutrition.

This specimen displays to a great degree those remarkable cavities in the substance of the dentine with which the tubes communicate, and which are obviously a modification of the tubular structure. They consist of variously formed *loculi*, having the same physical appearances under the microscope as the dentinal tubes; but their form is infinitely varied. Upon a superficial examination they may be mistaken for interglobular spaces,* from which, however, they are essentially distinct. (Plate I, Fig. 3.) The outlines are not circular, or semicircular, as with the interglobular spaces, nor have they the dense black

* Mr. Tomes long since indicated the distinction of the former from the latter. I do not agree with him that they are "not uncommon" "especially towards the outer surface of the dentine." In primary dentine I have much more frequently found them in the last, least regularly formed layers of the tissue, near the pulp-cavity.

opacity of the latter, when filled with air. Moreover, the outlines of the tube are seen to diverge, as they follow on the wall of the loculus; the cavities of the two are plainly common. The term "radiating cells," applied by Mr. Tomes to these loculi, appears to me to be hardly admissible, as they are found of every conceivable form, from long hollow cylinders, produced by the collateral fusion of many tubes, to circular, or stellate cavities, including every imaginable intermediate shape. I venture to designate them *dentinal loculi.*

It would be tedious to describe the different forms that these loculi display in this specimen. I have figured some of the most remarkable of them.

The account of this specimen would not be complete without observing, that the removal of the tooth (though accompanied with a violent paroxysm of neuralgic agony) was followed by a total cessation of pain. Pain never recurred. It may surely be concluded that the pressure of this adventitious growth had caused the neuralgic affection—the removal of the tooth being followed by a complete cessation of pain, and the adventitious growth being the only morbid appearance discoverable.

2. *Cancellated or Vascular Exostosis on the Fang of a Bicuspid Tooth.*

I am indebted to my friend Mr. Walter Jones, of Worcester, for the specimen from which these drawings are made, and upon which these observations are based. The tooth was a superior bicuspid; the crown and upper part of the fang were normal in form, but the lateral surface of the former exhibited caries. On the lower half of the fang there was a considerable incrustation of hypertrophied tooth-bone, not gradually increasing, but commencing suddenly at the centre of the fang, and covering the whole lower half. This exostosis exhibited an unusual appearance. Instead of the smooth dense structure, and unbroken, or, at most, undulating surface of the mass, usual in dental exostosis,* this specimen displayed a loose, porous,

* Ordinary exostosis of tooth-bone are too common and too well understood to merit a lengthened description in this place; but the above-mentioned friable exterior, opaque, and dead-white. In the accompanying figure of the tooth (enlarged two diameters, for the purpose of rendering it more intelligible) this porous surface is indicated (Plate II. Fig. 3.) Upon viewing the cut surface of the fang of the tooth, the crusta petrosa is seen to be extremely porous, and having, from the section of numerous and large vascular canals, a cancellated aspect. The openings of the cut canals are larger towards the outer surface, and the structure becomes more and more compact in passing from without, inwards. This can be seen with the naked eye. When a section is viewed with low powers of the microscope, large numbers of canals are seen, of various sizes, meandering through the substance of the tooth-bone; they are of very unequal size, and some of them differ much in diameter in different parts of their course. The canals are very erratic; diverging, converging, branching and uniting at every conceivable point, and in all directions. From this circumstance it was impossible to have a complete view of the canals for any great space, and they are observed to be cut across after pursuing a short irregular course. In the accompanying figure (Plate II. Fig. 4,) some of the canals are seen entering the cancellated openings; some have half their walls ground away, so as to present grooves and not canals; in others the canals are perfect. The size of these canals may be said to vary generally from $\frac{1}{1000}$th to $\frac{1}{100}$th of an inch in diameter.

Besides these canals, there are a considerable number of those dark interspaces which are occasionally seen in imperfect and faulty tooth-bone, and which are probably due to incomplete calcification. With higher powers, are observed numerous lacunæ, very irregularly scattered, and having no definite specimen exhibits histological characters, which are quite unusual. The common exostosis is a dense mass, without pores, or irregularities of surface, consisting of even coatings or chalcedone-like lumps, laminated, and with the laminæ parallel to the surface. It is rarely vascular, but when it does contain vessels, there are seldom more than one or two canals: when they do occur they are surrounded by true Haversian systems of tooth-bone. More often they are small *culs de sac*, containing small processes of periosteum.

relation in axis and direction to the course of canals near which they are situate—nothing in fact, after the fashion of Haversian systems. The lacunæ are mostly rather small, and are very generally surrounded by the clear lunated outline, considered by Messrs. Tomes and De Morgan to be the wall of the "*lacunal cell.*"

3. *Fatty Degeneration or Decomposition of the Tooth-pulp.*

There is a peculiar affection, to which the teeth, especially those with one fang, are liable, and which has been designated "*necrosis.*" In this condition, the organized union between the tooth-pulp and the subjacent tissues becomes severed; the pulp no longer receives a supply of blood, and perishes. The causes of this, which are usually mechanical, need not be alluded to in this place. When a tooth has become thus necrosed, the condition is usually followed by some local inflammation,* which requires the removal of the necrosed organ. In other instances, much less usual, the pulp remains in a dead condition, boxed up in the pulp cavity, without causing much inconvenience, and it is the changes which it, as well as the tooth itself, then undergo, that are the subject of this communication.

I recently had an opportunity of examining a tooth in which this had taken place. Upon opening the pulp-cavity, the pulp was seen of a very dark color, and of a soft, almost diffluent consistence. In small portions placed under the microscope I found, interspersed among an unintelligible debris, countless multitudes of minute molecules, highly refracting, and with a brilliant centre. The great similarity of these particles to fat or oil-globules, such as are seen in degenerate tissues, was at

* I have not here, gone into the history of these inflammatory results. It would be out of place. I may, however, briefly state, that they end usually in one form of alveolar or tooth abscess: either the common alveolar abscess, which points and opens at the side of the alveolus, at a point corresponding to the extremity of the fang of the tooth ; or fistulous abscess, forming a gutter in the alveolus, from the apex of the fang, to the edge of the gum. In the latter, this channel is indicated by an incrustation of green tartar on the side of the fang. I believe that in all cases the denudation of periosteum is secondary.

once apparent, and I demonstrated their identity by treatment with ether, when they instantly dissolved and vanished. These fat granules were mostly very small, few reaching the size of a blood-disc; the smaller ones, when diffused through water, had a brisk molecular motion, and thus seen, the object had a resemblance to the "molecular base" of chyle.

Not only did the tooth-pulp exhibit this fatty condition, but the dentine of the tooth appeared also to become permeated with fat. The tooth was, in this condition, of a darker color, and clearer than the normal structures, and this latter character was especially marked in the specimen from which these observations were made. It need scarcely be remarked that the white opacity of dentine is produced by the light-refracting tubes, which exist in multitudes in its structure, and which containing in life a thin serum, and in dried specimens, air, materials both of slight light-refracting power, as contrasted with the walls of the tubes and the intertubular tissue, prevent the transmission of light through the tissue, and reflect it of a dead white color. If, however, these tubes are filled with any highly refracting fluid, such as turpentine or oil, the tubes no longer prevent the transmission of light, and the structure becomes semi-transparent, and of a yellowish color.

Now this, it would be seen, was the change that had occurred in the clear dentine of the tooth in question.* I made several sections of this tooth near to, and at the neck; when ground down of the ordinary thickness for microscopical examination, they were extremely clear, and the dentinal tubes only appeared as faint radiating striations. Some of these specimens I then boiled in ether, so as to extract any fatty matters they might contain, and upon drying them, their characters were completely altered; the white opacity of the dentine was completely restored, and the dentinal tubes resumed their normal

*The clearness of this tooth, and I suspect of all such clear dead teeth, must not be confounded with the semi-transparent fangs of teeth which have produced, or been the subjects of irritation or inflammation. In these, the tubes are filled up with calcareous matter, especially towards their outer limits; moreover, the action of ether has no effect upon such clearness.

aspect. These conditions may be seen by viewing the specimens presented, one of which was mounted without alteration or treatment, the other was boiled in ether for some minutes.

This circumstance of the fatty change in a tooth-pulp that has ceased to receive nourishment, and in a decomposing state, appears to me to be interesting, not only as a new item in the pathology of the teeth, but as still more so in a general pathological sense, in relation to the curious subject, *fatty degeneration.* Here is a structure, the tooth-pulp, chemically of an albuminoid nature, *containing not one particle of fat;* its nutrition is suspended, and as a result, a considerable amount of fatty matter is developed.

### 4. *Warty Tooth.*

I have applied the term "warty" to a peculiar condition of tooth, in which the crown is more or less complicated and compound, not by the formation of cusps, but by the development of numerous papillary projections and villous foldings of surface into which all the proper elements of the tooth crown enter. Each papilla or villus being composed of a mass of dentine clothed by enamel, and containing within it, a pulp cavity or vascular canal; the whole mass together presenting an appearance not unlike a common tegumentary wart.

The tooth, which is especially the subject of this communication, and from which the accompanying figures have been taken, is a right upper lateral incisor; and for it I am indebted to Mr. H. Hamilton, of Poole. This tooth is represented in Fig. 1, Plate III, enlarged about two diameters to render its form and proportions more obvious. Upon consulting this figure it will be seen that the tooth was bent upon itself from before backwards, and when viewed sideways, presented an outline like the letter *f.* The crown was well formed, large, and somewhat compressed from before backwards: the root short and thick. In the front of the tooth, extending both above snd below the neck, was an irregular lobulated mass, the size of a horse-bean, of a brown color, and of a porous cancellated structure, and looking very like, (especially when seen *in situ,*) what one might imagine an exostosis from the edge of the alveolus denuded of periosteal covering. The surface was extremely irregular, exhibiting every species of complication—foldings, projections, pores, interspaces, and depressions. This abnormal growth evidently belonged to the tooth, and seemed intimately connected with, and to form a part of the anterior inferior portion of the crown.

To ascertain the real nature of the mass, as well as to determine its precise relation to the tooth, I made a vertical section of it and the tooth from before backwards. Up to a certain point I was tolerably successful; but, owing to the extremely friable nature of the adventitious growth, and from the fact that I accidentally ground too much from one side of it, some of the most telling and illustrative points of its structure were rubbed out and effaced.

The accompanying figure (Fig. 2, Plate III,) indicates the anatomical composition of this specimen as seen during the progress of the section; and, though rather diagrammatically expressed, it is, nevertheless, absolutely correct. There is necessarily much confusion in the appearances, as a section in any plane through a mass consisting of layers of different tissues, moulded in every conceivable complication of service and general arrangement, must obviously cut them in the most varied proportions and positions.

In the progress of the section I found that the pulp cavity gave off at its neck, anteriorly, a large canal, in a direction downwards and forwards. After a short passage this divided into three or more smaller canals; some of which were soon lost in a confused mass of dentine and enamel; while another could be traced, for a considerable distance in the center of the outer layer of the warty mass. On tracing, in the accompanying illustration, the most anterior of the smaller canals, it is seen to pass forwards and downwards. The canal is of pretty equal diameter throughout; and the same is the case with the lamina itself. At the bottom of the mass this canal ceases to be apparent within the lamina, from being ground out; but the lamina itself bends backwards, and from its upper surface, project, at this part, two small papillæ, of the same structure as the lamina, and, doubtless, containing originally a vascular canal each. These latter project into a sort of heart-shaped cavity—an apparent cavity caused by the involution of the surface, which originally had within it many papillary and folded processes.

This adventitious mass contains all the tissues which are normally found in a tooth crown—pulp-cavities in the form of vascular canals, on each side of which are layers of dentine; and beyond these again, coatings of enamel. The relation and proportion of these structures are very apparent in the lamina just described. On the outer surface is a layer of enamel not only covering the exterior surface, but that (produced by the involution) of the heart-shaped cavity within it. The dentine is somewhat thinner than the enamel; it presents the ordinary arrangement of parallel tubes, diverging from the pulp-cavity, and passing in curves to the enamel base. This is better shown in Fig. 3, which is not a diagram, but an exact portrait of a portion of the lamina, magnified forty diameters.

In the more confused portions of the section of this mass, those parts nearest the tooth itself, the structure is less intelligible. Vascular canals are only seen for a short distance of their course. The dentine is irregularly distributed in small quantities; and thick opaque enamel is very abundant. All this is indicated in Fig. 2.

The only other specimen I have seen, exhibiting in any way a similar structure, occurred in the practice of a distinguished metropolitan surgeon some six or eight years ago; and, indeed, of the exact nature of this specimen I cannot speak with absolute certainty, as I have had no opportunity of examining it with the microscope; and it is now some time since it was in my hands.

A young man, æt. 20, was admitted into a metropolitan hospital, having a mass resembling bone within the mouth, on the upper surface of the horizonal ramus of the lower jaw, immediately behind and in a line with the second molar tooth on the right side. The mass had gradually been rising in the mouth for some time, and was, when the patient was admitted into the hospital, as large as a small walnut; and, by its continued projection, had gagged open the mouth. To all outward appearances this mass very much resembled a piece of porous bone denuded of periosteum, and this, indeed, it was considered to be.

For the purpose of extirpating the mass, a portion of the angle of the jaw, in which it was implanted, was removed; and subsequently, to ascertain its anatomical structure, a vertical section was made through the whole. This at once disclosed the nature of the peculiar growth, and showed that it was in reality a malformed tooth; and that the supposed exostosis was the crown of the wisdom tooth in a state of warty complication. The fang of the tooth was almost normal, and was seen in the section to be implanted in an appropriate socket, and containing a single pulp-cavity; but the crown of the tooth was wholly malformed, and in no part indicated its nature as a tooth. It was not, as in my specimen, a warty mass superadded and attached to an obvious and unmistakable tooth-crown, but the whole was abnormal, and, until seen in section, unintelligible. The error in diagnosis was, therefore, not only natural, but almost inevitable.

Of the manner in which these malformed teeth are produced, there can, I should think, be but one opinion. Our knowledge of the laws which regulate the development of these organs is so complete, and the fact of their inability to undergo any subsequent change in outward form is so well established, that this malformation must certainly be considered as a *vitium primæ formationis*, and can only be explained by supposing the existence of a formative tooth-pulp, as warty and as complicated as the tooth subsequently developed from it.

I am not acquainted with any account of similarly malformed teeth; nor do I know of the existence of such specimens in any of our museums.*

* Since the above was written I have found in the Museum of Guy's Hospital, two teeth, (a superior lateral incisor and a superior dens sapientiæ,) exhibiting small warty excrescences.

### 5. *Hare Lip, associated with Double Cleft Hard Palate.*

These drawings were made, and this specimen obtained from a little boy, æt. 18 months, who came under the care of Mr. Hilton, at Guy's Hospital, in October, 1854. The child exhibited an unusually severe double hare-lip; and, behind it, a deep double cleft in the intermaxillary bone. The cleft in the lip passed from the alæ nasi vertically to the edge of the lip on either side. The central flap, thus produced, was tilted forwards by the mass of bone behind, so as to cause an interval of about three-quarters of an inch between it and the lateral portions of the lip. The intermaxillary bone was very deeply cleft in lines corresponding with the fissures of the lip; and the central mass, thus laterally separated from the rest, projected nearly horizontally forwards (Plate II, Fig. 1.) This mass of bone, by its great size and projection, (thus separating the three portions of the lip so widely,) was such a complete barrier to a successful operation for the cure of the malformation, that its removal was determined on as a preliminary step. The rest of the operation was then successfully performed, and the child has now a very fair upper lip.

The mass of bone removed was committed to me by Mr. Hilton, for the purpose of its structural examination. It is of a quadrilateral form, about eight lines long, eight broad, and six or seven thick. A considerable portion of its surface (that which is about its anterior half, and the whole of its sides) is covered with ordinary gum. The posterior surface exhibits cancellated osseous tissue where it was cut from the upper jaw. On the front upper surface of the gum is a vertical raphé, similar to the one usually found passing from the frænum of the upper lip, to the point of gum in the interval between the two superior central incisors. The inferior or palatal surface of the gum displays a triangular ruga, with its apex forward, similar to the one normally seen in the front of the palate, immediately behind the central incisors. The left temporary central incisor has just pierced the gum; and the right, far advanced, can be felt immediately beneath the surface. From the general aspect of this specimen, I was inclined to consider it as the incisive or intermaxillary bone, separated by a lusus naturæ from the maxilla proper; a section of it, however, throws some doubt upon that opinion, as the rudiments of the lateral incisors are not contained in it, and such an idea is only tenable by imagining the laterals altogether suppressed.

Upon attempting to make a section, it was found that ossification had been so complete, and the mass was so hard, that it could not be accomplished without probable injury to its internal structure, and I, therefore, decalcified it by immersion in dilute hydrochloric acid for a considerable time; it was then accomplished with ease. With a sharp thin knife I cut the mass vertically, and from side to side, commencing immediately behind the incisor teeth. The section was a happy one, and displayed at one view the whole internal structure. The cut surface, (Fig 2, Plate II,) exhibits the imperfectly developed and rudimentary germs of the superior central incisors of the temporary and permanent set; the former advanced in development, and the latter in a very early condition. They are all surrounded by a capsule of bone, and each separated from the other by crucial laminæ intersecting them, consisting of a horizontal lamina, dividing the temporary from the permanent teeth, and a vertical, separating the right from the left; the latter being slight, and partially divided by a fissure, the suture between the two incisive bones. The four osseous loculi thus produced are perfectly and beautifully symmetrical; and their contents are fully exposed. The two superficial ones exhibit the fangs of the rudimentary teeth obliquely cut across; the germs of the permanent teeth remain uninjured, and *in situ* within the deeper seated loculi.

The structural arrangement of this section will be best understood by reference to the accompanying figure, (Fig. 2, Plate II.) The letter *a* indicates the left central incisor tooth, which has pierced the gum; and *á*, the right, still covered by the gum. The sections of these teeth exhibit a mere ring of dentine, and an enormous pulp-cavity, arising from the very youthful and incomplete development of the teeth. Beneath these loculi are the two others, containing the very young germs of the permanent central incisors. These are in a very rudimentary condition, the pulps being only slightly capped with a thin layer of dentine.

The chief points of interest in this specimen are the entire symmetry of the malformation, the perfect coincidence of the clefts in the lip, the fissures in, and the horizontal projection of, the central isolated portion of the jaw; added to this it is remarkable that the soft palate was not divided, and the uvula was single.

---

### DESCRIPTION OF PLATE I.

The figures illustrate Mr. James Salter's case of dentine-excrescence in the pulp-cavity of an incisor tooth, (*see* p. 14.)

Fig. 1. *Dentine-excrescence* in the pulp-cavity of the left superior central incisor. Enlarged 2 diameters.

Fig. 2. Vertical section of the excrescence. Magnified 30 diameters.

Fig. 3. Three variously formed "dentinal loculi." Magnified 150 diameters.

### DESCRIPTION OF PLATE II.

Figures 1 and 2 illustrate Mr. James Salter's case of double hare lip associated with double cleft palate, (*see* p. 26.)

Fig. 1. Head of the child showing the parts as they appeared before an operation was performed.

Fig. 2. Vertical section of the central projecting portion of the upper jaw after its removal. Enlarged about 2 diameters.

*a*, Left superior central incisor, having just pierced the gums, with large pulp-cavity, containing the pulp.

*á*, The right tooth covered in by gum.

*b*, *b*, *b*, *b*, Mucous membrane of the mouth (gum) covering the bony surface.

*c*, *c*, Cut surface, where the bone was attached.

Figures 3 and 4 illustrate Mr. Salter's description of a cancellated or vascular exostosis, (*see* p. 18.)

Fig. 3. Superior bicuspid tooth; the fang partly covered by vascular or cancellated exostosis. The line indicates the plane of the section. Enlarged 2 diameters.

Fig. 4. Section of the same, exhibiting numerous erratic vascular canals, cancellated openings, irregular insterspaces, and minute lacunæ. Magnified 48 diameters.

### DESCRIPTION OF PLATE III.

The figures illustrate Mr. James Salter's description of a warty tooth, (*see* p. 22.)

Fig. 1. Right upper lateral incisor, with warty projection from its anterior surface. Enlarged 2 diameters.

Fig. 2. Diagrammatic drawing of vertical section of the same, exhibiting the general structure of the mass.

Fig. 3. One of the laminæ of the warty mass, displaying the exact proportion and relation of the structures entering into its formation:—the outer layers of enamel, the small central vascular canal (pulp-cavity,) and the intermediate dentine. Magnified 40 diameters.

PLATE I.

## Figures and Tables

**Figure f1:**
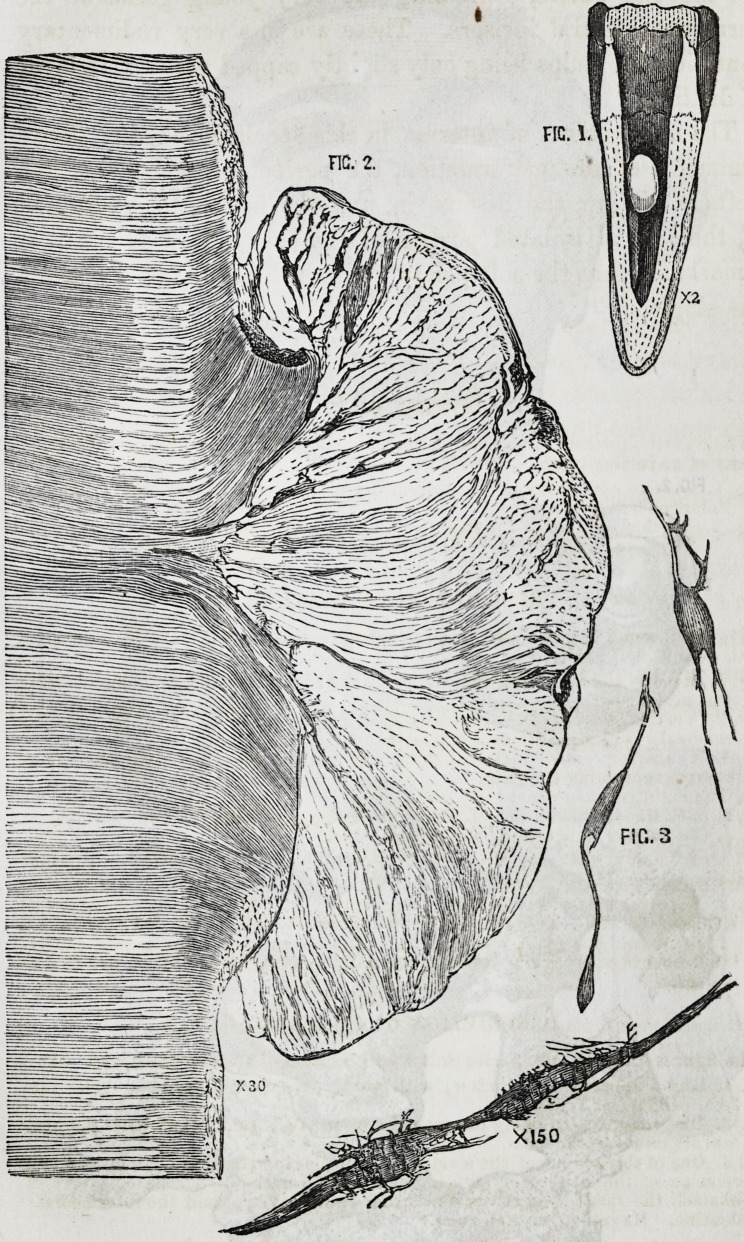


**Figure f2:**
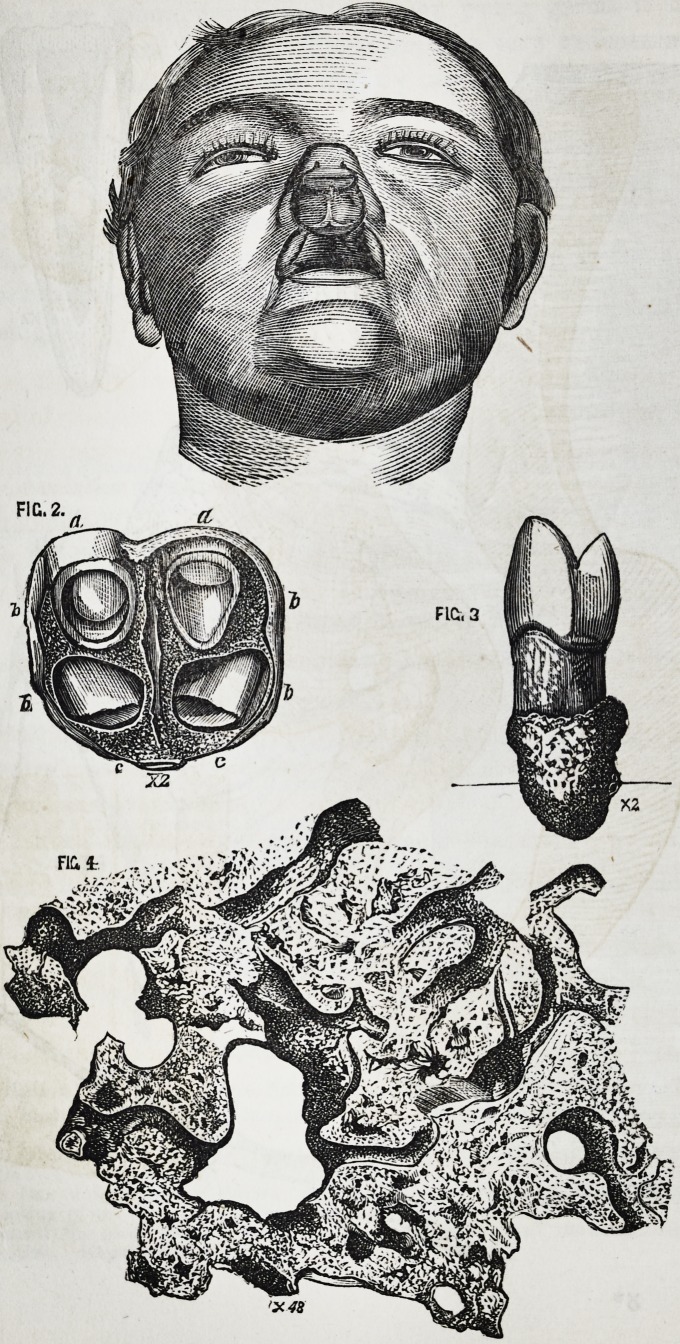


**Figure f3:**